# Recoverable Extensional Flow of Polymer Melts and Its Relevance for Processing

**DOI:** 10.3390/polym12071512

**Published:** 2020-07-08

**Authors:** Helmut Münstedt

**Affiliations:** Department of Materials Science and Engineering, Friedrich-Alexander-University Erlangen-Nuremberg, D-91058 Erlangen, Germany; helmut.muenstedt@fau.de

**Keywords:** polymer melts, recoverable elongation, entrance flow, short dies, extrudate swell, filled polymers, polymer blends, shrink films, extrusion processes

## Abstract

While the uniaxial elongational viscosity is widely investigated, and its relevance for processing is described in the literature, much less has been published on the recoverable extensional flow of polymer melts. This paper presents a short overview of the dependencies of the recoverable elongation on the molecular structure of a polymer, and on some experimental parameters. Its main focus lies on the discussion of processing operations and applications that are largely affected by the elastic components of elongational flow. The recoverable portions of stretched films are considered, and the exploitation of the shrinkage of films, due to the recovery of frozen recoverable deformations, and its role for applications are addressed. The analysis of measurements of velocity fields in the entry region of a slit die and results on the determination of the recoverable elongation from uniaxial experiments, according to the literature, lead to the conclusion of dominant elastic extensions. Considering these facts, the assumptions for Cogswell’s widely used method of determining elongational viscosities under processing conditions from entrance flow are not realistic. As examples of a direct application of extrudate swell from short dies for processing, pelletizing and fused deposition modelling within additive manufacturing are addressed. The special features of extrudate swell from short dies, and uniaxial recoverable elongation for a polymer filled with rigid particles in comparison to an immiscible polymer blend, are presented and discussed.

## 1. Introduction

The rheological properties of polymer melts have shown their importance for the assessment of processing polymeric materials over the years. In particular, the shear viscosity has been widely used to get a deeper understanding of processing operations like extrusion or injection molding, and the dependence of viscosity on rate and temperature has become the base of material functions applied in numerical descriptions by special models. Moreover, polymers are processed by well-known techniques, where the deformation mode is predominantly extensional. Examples are fiber spinning, blow molding, and film manufacturing. These processes are performed at highly nonlinear conditions, and that is the reason why the corresponding rheological behavior cannot be derived from shear rheology as has been discussed in [1], for example. Special experimental methods to measure elongational properties of polymer melts were developed starting in the 1970s. They are comprehensively described in textbooks and the literature on rheology. 

The most exciting feature of elongational flow is so-called strain hardening, which describes the increase of the elongational viscosity as a function of extension or time of deformation, respectively, far beyond the shear viscosity for some polymers. It is obvious that such a behavior may influence the processing of polymers and end-use properties of processed items, and it was shown how the uniformity of deformation, in particular, is favorably affected by strain hardening. The state of the art in this field was reviewed rather recently [2]. 

Polymer melts are viscoelastic, and viscosity is only one part of their rheological properties. In pioneering experiments, the recoverable portions of a low-density polyethylene (LDPE) were quantitatively determined in uniaxial extension at various experimental parameters, and it was shown that the elastic deformation may attain rather high values [3]. In spite of this remarkable effect, the role of elasticity in processing operations with dominating elongational flow has been much less discussed than that of viscosity. Thus, the intention of this paper is to address this field in more detail. 

## 2. Methods 

### 2.1. Experimental Devices

One reason for the limited number of publications on recoverable elongation is the scarce laboratory equipment to measure this quantity. Five experimental devices for investigating extensional properties of polymer melts were developed over the years. An overview of their principles and functions can be found in textbooks on rheology (see [1,4,5], for example). Only three of them enable measurements of the recovery of elongated samples, but they are not available commercially. In the pioneering work by Meissner, a cylindrical rod 800 mm in length is stretched between two pairs of rotating clamps. At any state of deformation, the sample can be cut by scissors into parts whose recovery may be measured [3]. Based on the principle of rotating clamps, the Rheometrics Extensional Rheometer (RME) was built [6]. After cutting the rectangular sample at one end in any state of deformation, its time-dependent recovery can be monitored by a camera. A versatile rheometer for investigating the elongational behavior of polymer melts in extension is the Münstedt Tensile Rheometer (MTR) [7,8]. Here, a cylindrical sample suspended in a silicone oil bath is vertically stretched via an electronically controlled driving unit. Measurements at a constant elongational rate or constant actual stress can be performed. The equilibrium elastic deformation is determined from the length of the sample frozen by lowering the oil bath and annealing the sample outside the rheometer in silicone oil. Alternatively, the time dependence of recovery can be determined in good approximation by the MTR, making use of the method of stepwise sample unloading sketched in [9]. The other two instruments applied today in extensional rheology, namely the extensional rheometer after Sentmanat (SER) [10] and the filament stretching rheometer (FSR) [11], can be used only to determine elongational viscosities and, thus, do not provide any information on elastic properties.

Two experimental modes find applications in extensional rheometry: measurements at constant elongational rate (stressing experiments), and those at constant actual stress (creep experiments). Creep experiments are not so widely used, because keeping the actual stress constant for highly elongated samples requires a sophisticated control of the driving unit. However, in creep a steady state of deformation is reached at smaller total extensions than in experiments at constant elongational rates as discussed in [1]. Such a behavior is obviously of advantage when a good uniformity of sample deformation is required for accurate measurements of elongational properties, particularly in the steady state [12]. The MTR is especially suited for creep experiments.

### 2.2. Evaluation of Experimental Results

**Figure 1:**
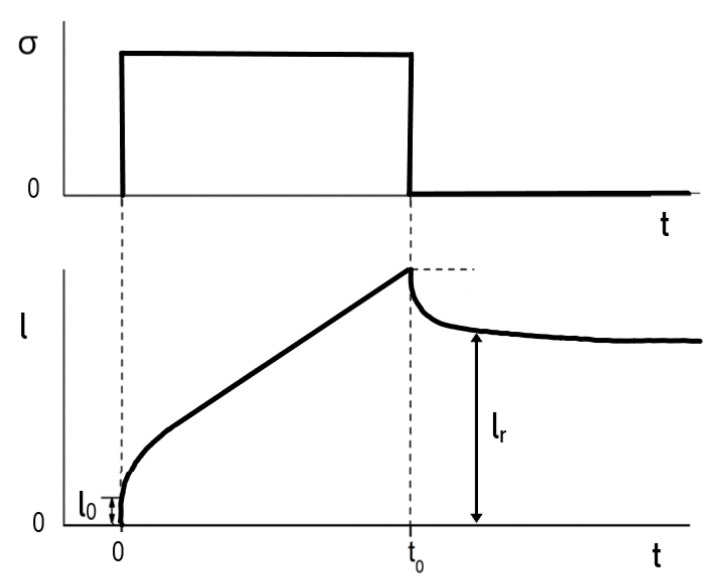
Tensile stress σ and sample length l as functions of experimental time t in a schematic creep recovery experiment on a polymer melt.

Figure 1 sketches a creep recovery experiment as it can be performed with the MTR, for example. At the time *t* = 0 a constant tensile stress σ is applied to a sample with the initial length $l_{0}$. At $t_{0},$ the stress is set to zero. During the creep time $t_{0}$ the sample has attained the length l from which it gradually recovers to the length $l_{r}$ when removing the stress and reaches the constant length $l_{rs}$ after some time, which represents the equilibrium state after total recovery. In extensional rheology of polymer melts the total deformation is usually expressed by the so-called Hencky measure as

(1) $$\varepsilon_{H}\;=\ln\;l/l_{0}=\ln\;\lambda \;$$

with λ being the stretching ratio. The recoverable strain follows as

(2) $$\varepsilon_{r\;}=\ln\;l/l_{r}=\ln\;\lambda_{r}$$

where λ_r_ describes the recoverable part of the stretching ratio. For the matter of convenience, the index “H” is omitted, because only the Hencky measure is used throughout this paper. From Equation (1) the elongational rate follows according to

(3) $$\dot{\varepsilon \;}=d\varepsilon /dt\;=v/l$$

with v=dl/dt.

The material-specific quantities obtained from experiments in elongation are elongational viscosity µ and elongational compliance D defined as

(4) $$\mu =\sigma /\dot{\varepsilon }$$

and

(5) D=ε/σ

From the recoverable elongation $\varepsilon_{r}$ the recoverable compliance follows according to

(6) $$D_{r}\;=\varepsilon_{r}/\sigma$$

and for the recoverable compliance in the steady state one gets from the steady-state recoverable elongation $\varepsilon_{rs}$

(7) $$D_{rs}=\varepsilon_{rs}/\sigma$$

More detailed information on the definition of physical quantities following from elongational rheology can be found in corresponding textbooks (for example [1,4,5]).

## 3. Results

### 3.1. General Features of Recoverable Elongation 

#### 3.1.1. Dependence on Experimental Parameters

**Figure 2:**
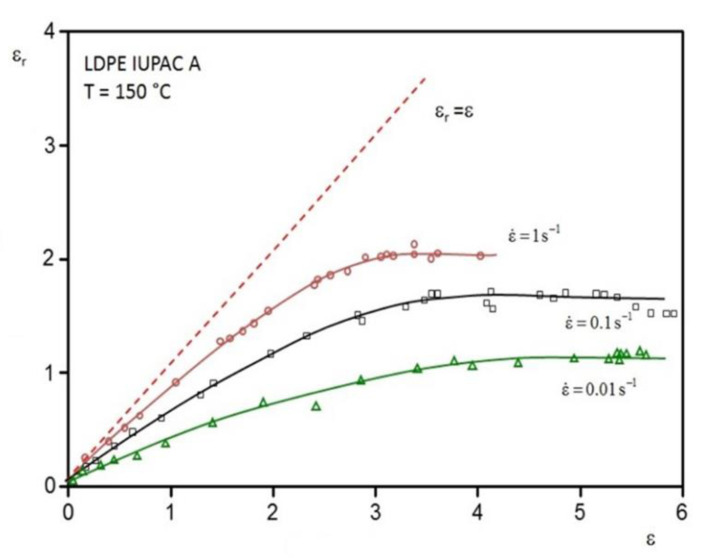
Recoverable elongation $\varepsilon_{r}$ as a function of the total elongation ε in experiments at constant elongational rates $\dot{\varepsilon }$ and a temperature of 150 °C for a low-density polyethylene (LDPE). The experiments were performed with the Meissner oil bath rheometer. Each symbol corresponds to one sample stretched to a definite total elongation and then recovered after cutting into pieces [13].

Similar to viscosity, elastic properties are functions of experimental time. This feature becomes obvious from Figure 2, which presents measurements of the recoverable elongation $\varepsilon_{r}$ in dependence on the total extensional strain ε by experiments at constant elongational rates. It is evident that $\varepsilon_{r}$ increases first with ε, which is proportional to the duration of the experiment in this mode, and then reaches a plateau value $\varepsilon_{rs}$. The plateau values are higher the larger the elongational rates or the corresponding tensile stresses, respectively. The broken line marks a state at which the elongation is completely recoverable as in the case of rubber, for example. Such a rubbery behavior is more closely approached at smaller elongations or deformation times, respectively, and higher elongational rates. It may be noted that at the conditions chosen for the measurements in Figure 2, $\varepsilon_{r}$ is larger for higher elongational rates over the whole range of deformation.

**Figure 3:**
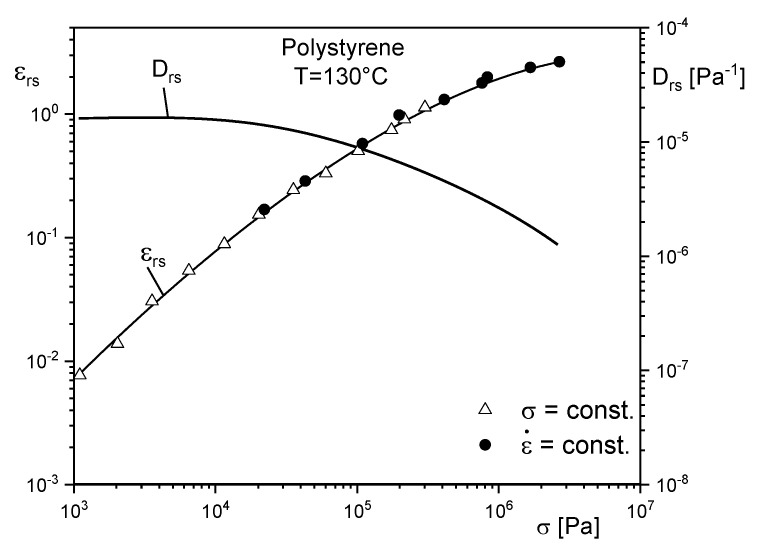
Recoverable elongation $\varepsilon_{rs}$ and recoverable compliance $D_{rs}$ in the steady state as functions of tensile stress from creep and stressing experiments for a polystyrene at 130 °C [7].

The stress dependence of the steady-state recoverable elongation $\varepsilon_{rs}$ is exemplarily represented on polystyrene in Figure 3. Data from measurements at constant stresses and constant elongational rates coincide, as should be expected, because properties in the steady state are not dependent on the way it is attained. Within three decades of stress, $\varepsilon_{rs}$ increases by about two decades. At small stresses, an approximately linear relation between $\varepsilon_{rs}$ and σ is found, which is obviously reflected by the stress independency of the steady-state recoverable compliance $D_{rs}$ in this range defined by Equation (7). At higher stresses, $D_{rs}$ becomes nonlinear and decreases with σ.

**Figure 4:**
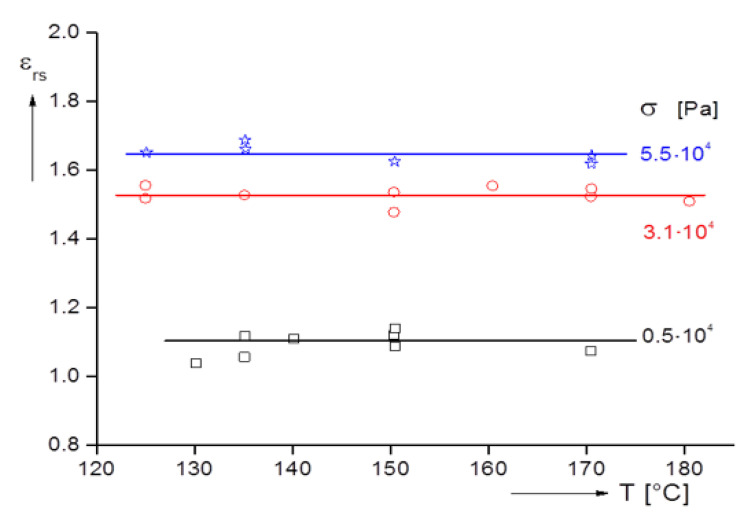
Steady-state recoverable elongation $\varepsilon_{rs}$ as a function of temperature for an LDPE at different tensile stresses σ up to the steady state indicated by a time-independent elongational rate [13].

As another important experimental parameter, the influence of the temperature on the recoverable elongation is presented in Figure 4 for an LDPE. For the creep experiments performed at different stresses, it is obvious that the steady-state values are independent of temperature at least in the range between 130 and 180 °C. Additionally, the increase of $\varepsilon_{rs}$ with stress corresponding to that with elongational rate in Figure 2 becomes obvious. 

#### 3.1.2. Dependence on Molecular Structure

Reliable measurements on the influence of molecular structure on recoverable elongation are still less frequent than those on the effect of experimental parameters. In the linear range of deformation, conclusions could be expected from measurements in shear according to the relation

(8) $$D_{rs}^{0}=J_{e}^{0}/3$$

where $J_{e}^{0}$ stands for the steady-state recoverable shear compliance in the linear range of deformation. This quantity is easier to determine than its pendant in elongation and, thus, many results of the dependence on molecular structure are known (see [9], for example). Obviously, these relations cannot be transferred to the highly nonlinear regime, dominating elongational flow, because theories linking these two regimes are not available. 

**Figure 5:**
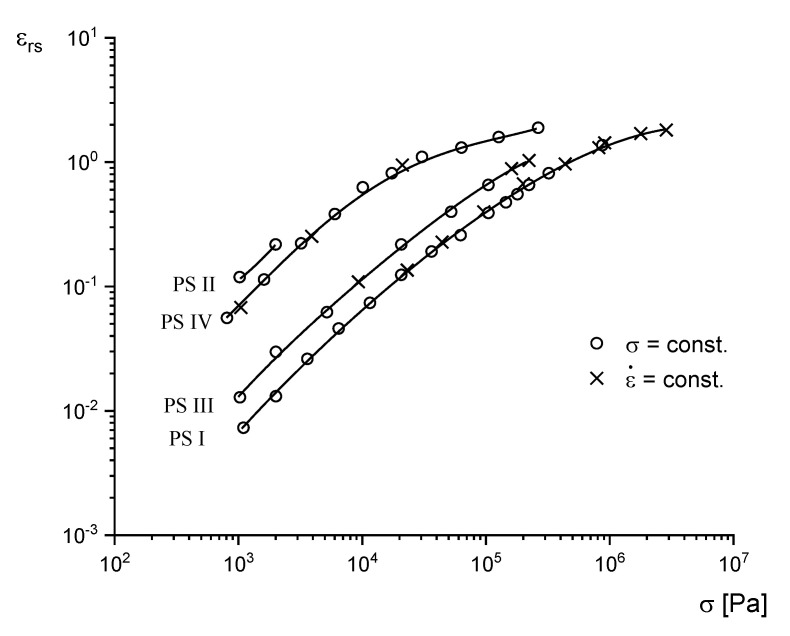
Steady-state recoverable elongation $\varepsilon_{rs}$ as a function of tensile stress σ for polystyrenes with different molar mass distributions. PS I is anionically polymerized with a very narrow distribution, PS II is an anionically polymerized sample with a distinct high molar mass tail, and the anionic PS III is marked by a tail extending to lower molar masses [15]. PS IV is a commercial product with a pronounced broad distribution. The results from measurements at constant stress and constant elongational rate are in good agreement.

In [14], it is reported that the steady-state recoverable elongational compliance measured in the linear range of deformation $D_{rs}^{0}$ for narrowly distributed polystyrenes is independent of the weight average molar mass for molar masses distinctly above the critical value for entanglements. From Figure 5, conclusions can be drawn with respect to the influence of the molar mass distribution on recoverable elongation. The results are presented for four polystyrenes [15], which, as is well known, have a linear structure according to their way of polymerization. For all the measurements presented in Figure 5, $\varepsilon_{rs}$ is considered at a distinct stress, increases from PS I over PS III to PS IV, and this enhancement can be related to a broadening of the molar mass distribution, because the molar masses of these samples do not have an influence on the recoverable elongation. The surprisingly large data of PS II is due to the significant high molar mass component of this material as discussed in [9]. All materials exhibit a linear range of deformation, where $\varepsilon_{rs}$ is proportional to *σ* and, consequently, the steady-state compliance is constant for each sample. From these results, it can be said that the steady-state recoverable elongation at a distinct stress and, therewith, the steady-state recoverable compliance increase with broadening the molar mass distribution, or introducing a high molar mass component. 

**Figure 6:**
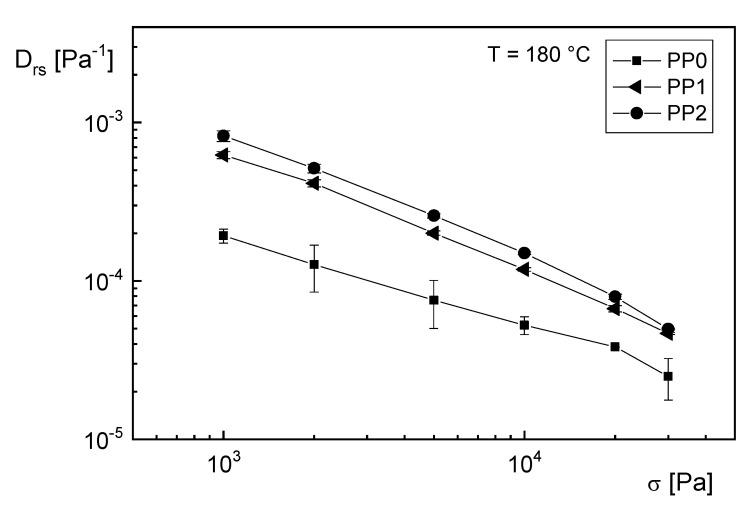
Steady-state recoverable compliance $D_{rs}$ as function of tensile stress σ for the linear commercial polypropylene PP0 and samples electron beam irradiated with 1 kGy (PP1) and 2 kGy (PP2) [9].

The effect of long-chain branching on the recoverable compliance can be concluded from Figure 6. Here, $D_{rs}$ is presented as a function of tensile stress σ for a commercial linear polypropylene (PP0), and samples treated with 1 and 2 kGy of electron irradiation (PP1 and PP2), which generates long-chain branching [16]. The higher values of $D_{rs}$ for PP1 and PP2 in comparison to PP0 can be assigned to the long-chain branches, because the polydispersity indices of the irradiated samples are smaller than that of PP0 [9].

From the examples in Figure 5 and Figure 6, it may be concluded that the recoverable elongation, and there with the recoverable elongational compliance measured at the same conditions in the nonlinear range of deformation, increase by broadening the molar mass distribution or introducing long-chain branches. Quantitative descriptions have not been established up to now and, thus, the influence of molecular structure on elastic effects in elongation and, moreover, its relevance for processing have to be restricted to qualitative discussions. 

### 3.2. Recoverable Elongation and Its Function in Manufactured Items

In processing operations, the recoverable portions of elongational flow may come into play in different ways. The recoverable amount remaining in the fabricated item depends on the height of elastic deformation generated, and the effect of possible recovery processes during the forming process before the melt solidifies. In fiber spinning, flat film drawing, and tubular film extrusion the melt is frozen under tension, and the recoverable deformation stored is equal to that directly created during processing. Elastic properties of this kind and their exploitations are discussed in the following section.

Another effect of recoverable elongation has to be considered for processing operations in which elongation of the melt is induced somewhere in the processing chain, but retardation is possible for the recoverable portion. Such a behavior is found in all the operations where the melt is getting stretched by flowing through narrowing ducts and subsequently becomes able to recover in the wider portion of a flow channel with diminishing tensile stresses. These features can be met in nearly all extrusion and injection molding processes, and become particularly manifested in the swell of extruded parts. It should be mentioned, however, that in many tools, elastic effects from elongation are superimposed by those from shear. Thus, quantitative descriptions of the influence of recoverable elongation on extrudate swell are very difficult in general, but even qualitative considerations give insights into interesting features of processing. This topic is discussed in Section 3.3.2.

#### 3.2.1. Recoverable Elongation of Stretched Films 

**Figure 7:**
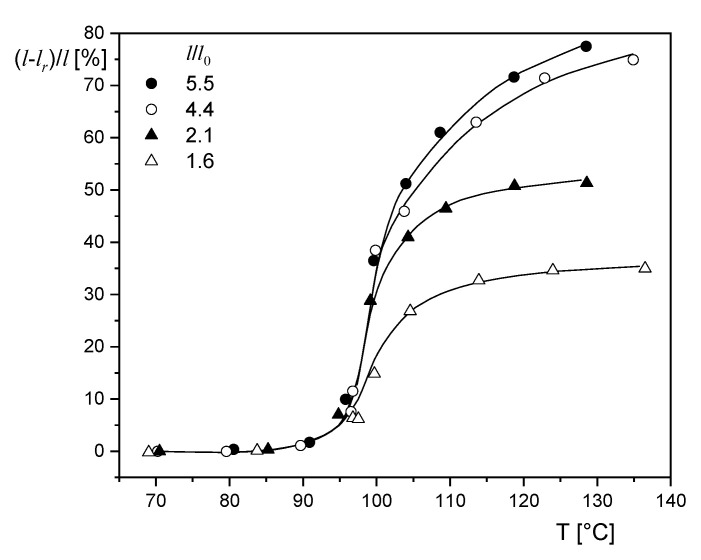
Shrinkage ratio $\left(l-l_{r}\right)/l$ as function of the annealing temperature T of polystyrene films previously stretched to different draw ratios $l/l_{0}$ at 130 °C and subsequently precipitated. The annealing time was 15 min [17].

Because of the viscoelastic nature of polymer melts the development of the elastic deformation and, consequently, its recovery is a function of time. This time dependence is determined by a spectrum of retardation times, which are affected by internal parameters like the molecular structure and external quantities as the temperature (see textbooks on rheology, e.g., [1,4,5]). An example from the literature is presented in Figure 7. According to [17], pressed polystyrene sheets were stretched at 130 °C and a rate of 4%/min up to different draw ratios $l/l_{0}$, and then precipitated. Subsequently, the stretched sheets were heated for 15 min at different temperatures below and above the glass temperature of 90 °C, and their lengths $l_{r}$ after recovery measured, from which the shrinkage ratio $\left(l-l_{r}\right)/l$ was determined. As expected, below the glass temperature the recoverable elongation is negligible, but it increases at higher temperatures and attains plateau values for the two lower draw ratios within the annealing time chosen at 15 min. This time is not long enough to reach the maximum shrinkage ratios for the samples prestretched up to the higher draw ratios obviously generating larger portions of recoverable deformation. 

**Figure 8:**
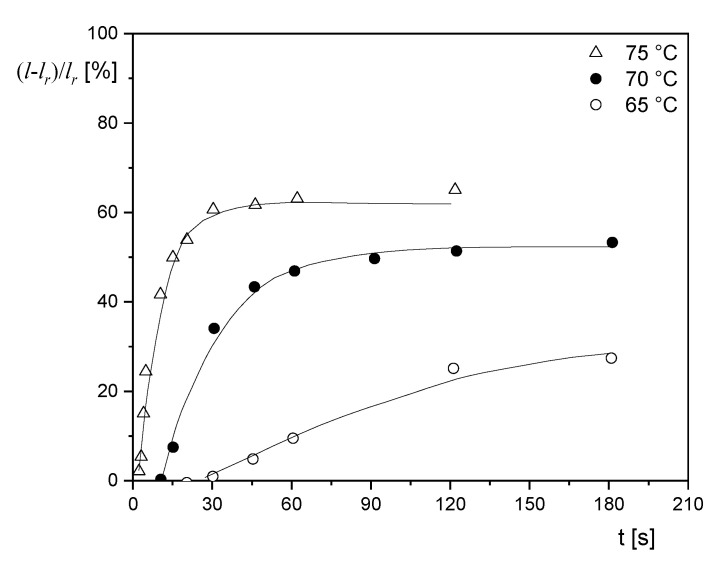
Shrinkage $\left(l-l_{r}\right)/l_{r}$ as a function of annealing time t at various temperatures for an extruded film from a polyethyleneterephthalate/glycol copolymer (PETG) uniaxially prestretched at 85 °C by a factor 3 before cooling. The full lines represent single exponential functions, according to [18].

Another example of the recovery of the elastic portion in stretched films is shown in Figure 8 for a polyethyleneterephthalate/glycol copolymer (PETG), which is commonly used in packaging [18]. The extruded PETG films of 50 µm were uniaxially prestretched at 85 °C by a factor of 3. The recovery at different annealing temperatures was measured as a function of time. The recovery rate distinctly increases with temperature. At 75 °C, a plateau value of shrinkage is attained after about 30 s, while at 65 °C, the steady state of shrinkage is far away, even after 180 s. As can be seen from Figure 8, the time, after which a measurable recovery occurs, significantly increases with a decreasing annealing temperature. 

#### 3.2.2. Shrink Films

The recovery of frozen portions of elastic deformation by heating is the base of shrink films, which are used in packaging. Semi-crystalline polymers play the dominant role in this field, because of their preferable end-use properties like tear strength and puncture resistance. For most of the applications, a homogeneous shrinkage of the film is desirable, thus, a uniform deformation in machine direction (MD) and the direction perpendicular to it (TD) is requested. Commonly for tubular films, the blow ratio in MD is distinctly more pronounced than in TD, leading to a non-uniform shrinkage. Therefore, special processing techniques are applied to produce strong biaxial deformations. One of them is the so-called double-bubble process, which has been widely described in the patent literature because of its commercial importance. The method is sketched in [9], and an example of a laboratory device given in [19]. 

The majority of shrink films is produced today from flat films. Two techniques are in use to generate biaxial deformation. In the sequential process, the extruded sheet drawn by a chill roll is reheated to temperatures somewhat below the melting point of semi-crystalline polymers, and longitudinally stretched in MD between rolls rotating with different velocities. Then the film is gripped by clips, which move on diverging rails and elongate it in the transversal direction TD. This biaxial deformation is frozen by cooling below the crystallization temperature. In the simultaneous process, the extruded film is stretched by clips moving on diverging rails with increasing velocities. In this way, the film is deformed in MD and TD at the same time. Each clip is driven by a linear motor, which can be controlled separately. Thus, the deformation becomes very variable, and its recoverable portion can be fixed by decreasing the temperature. Consequently, films with different shrinkage potential in MD and TD can be manufactured. Polymer materials based on polypropylene or polyethyleneterephthalate are very suitable for manufacturing biaxially stretched films by this technique. 

Shrink films have found wide applications in packaging because they are easy to use. The foils are wrapped around the item to be packed, and then heat is applied in a heating tunnel or locally by a heat gun. The film shrinks, due to the release of the frozen deformation, and tightly encloses the shape of the goods. Depending on the recoverable elongation generated and the retardation times of the material, only short exposure times to heat for the shrinkage required may be necessary and, thus, even heat sensitive goods can be packed with shrink films. The great spectrum of film properties provided by the various kinds of suitable polymeric materials, and their differences in the molecular structure are the base for the versatility of applications. Examples are the protection of books and boxes, and the overwrap of cartons and beverage cans. Even pallet loads are secured by shrink films, and applications can be found for the protection of new vehicles and machined parts. 

The use of heat induced shrinkage is not restricted to films, however. Heat-shrinkable tubes have found applications in wiring. For this purpose, an extruded tube is slightly cross-linked by electron irradiation, to increase the elastic portion of any deformation and improve the temperature dependence of mechanical properties. Then, the tube is expanded at a temperature around the melting point and, subsequently, the deformation frozen by rapid cooling. Surrounding a wire to be insulated, for example, the tube reheated by a heat gun tends to shrink to its initial diameter, and is pressed onto the wire surface. Contractions between half and one sixth of the widened diameter can be obtained and, thus, significant pressure forces can be generated. 

This technique is also used for a repair of broken plastic pipes by connecting the two ends with a suitable heat-shrinkable part. Shrink sleeves mainly from polyolefins are applied for the protection of steel pipes particularly at their welding spots where the usual plastic sheath is removed. The sleeves consist of extruded sheets, which are cross-linked and prestretched. They are wrapped around the particular part of the pipe and then shrink-fitted by local heating. 

These examples throw a light on how the recoverable strain of a polymer material generated by processing can be used for interesting technical applications.

### 3.3. Recoverable Elongation and Extrudate Swell

In addition to the apparent role of recoverable elongation in the applications discussed above, elongational flow is present during many processing operations without being directly obvious. For example, a polymer melt is stretched, when it flows through narrowing ducts and that can be found in many tools used in extrusion or injection molding. Due to their often complex geometry, the flow fields are difficult to assess or even to measure. These deficiencies are still more evident when the elastic portions have to be described that are inherent to viscoelastic fluids. Pioneering investigations of various components of the flow of polymer melts became possible at the beginning of the 1980s, with the commercialization of the laser-Doppler velocimetry (LDV) [20]. Further developments of this technique initiated comprehensive measurements of flow fields in the entrance and exit regions of a rectangular die, as well as of the features of the flow patterns inside [21,22,23]. Of special interest, with respect to the topic of this paper, are the elongational components, and particularly their elastic portions, which occur at the entrance and the exit of a die. Although a rectangular slit or a circular capillary show a simplified geometry in comparison to a real processing tool, results from this kind of laboratory experiments may support an at least qualitative understanding of some aspects of processing. 

#### 3.3.1. Entrance Flow

**Figure 9:**
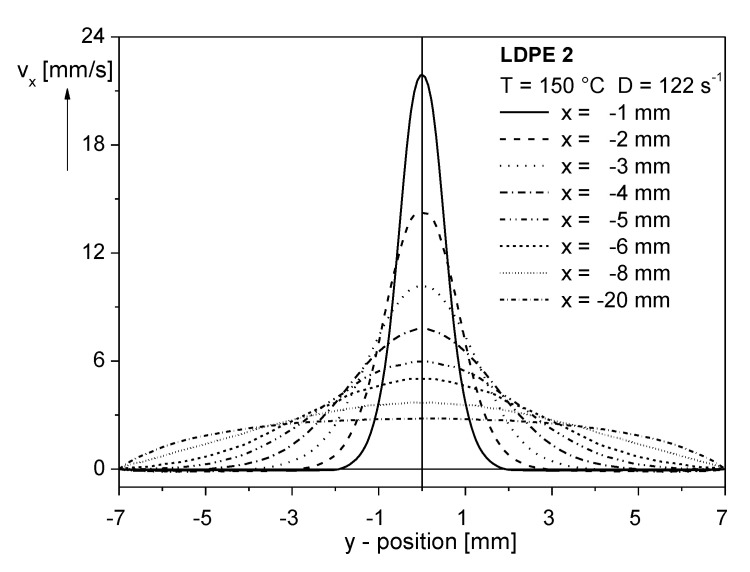
Velocity components $v_{x}\left(y\right)$ in the quadratic reservoir of a slit capillary with the height 1 mm at different positions x from the entrance plane for an LDPE at 150 °C and an apparent shear rate of D= 122 s^−1^. The measurements were carried out by laser-Doppler velocimetry (LDV) [21].

As it is well known, the entrance flow from a reservoir into a capillary may attain a complex feature, because of secondary flow phenomena occurring for some polymer melts with a long chain branched molecular structure (e.g., [1,21]). The most dominant features, however, are the velocity components in the direction of flow, which are exemplarily shown for a low-density polyethylene (LDPE) in the quadratic reservoir connected to a slit capillary with a height of 1 mm and a width of 14 mm (see Figure 9) [22]. At x = −20 mm, which means 20 mm above the entrance plane, $v_{x}$ is comparably small, and does not change much over the width of the reservoir in a *y*-direction perpendicular to the flow direction. By approaching the entrance plane, $v_{x}$ increases significantly, and its distribution along the y—axis becomes less uniform. For example, along the channel axis at $y=0,\;v_{x}$ at x = −1 mm, is eight times higher than at x = −20 mm, indicating a strong velocity gradient or elongational rate, respectively. 

**Figure 10:**
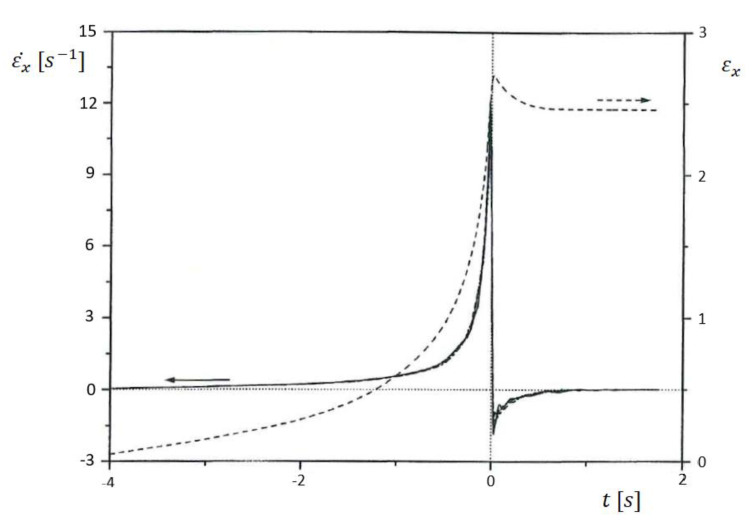
Elongational rate $\dot{\varepsilon_{x}}$ and total elongation $\varepsilon_{x}$ along the channel axis a function of time for a commercial LDPE extruded through a slit die with a reservoir of 14 mm squared and a capillary with a height of 1 mm and a width of 14 mm at 150 °C and an apparent shear rate of D = 100 s^−1^ [24]. The time was set to zero at the slit entrance.

In order to get an idea of the elongational rates and total elongations occurring in the entrance region of a slit die with the geometry described above, for a commercial LDPE, these quantities are plotted in Figure 10 exemplarily as a function of time [24]. As can be seen, the elongational rate steeply increases by approaching the entrance plane, and attains small negative values just behind the inlet, before it becomes zero in the slit, where shear is dominant. The elongational rate passes a wide range within some few seconds reaching 12 s^−1^ at its maximum just around the slit entrance. Even during this short time of flow, a total Hencky strain of 2.5 is attained, as follows from Figure 10. Most of the elongation is exerted during some few tenths of a second at the high elongation rates. Taking the results from Figure 2 into account that the recoverable portion of stretching is larger the higher the deformation rate and the shorter the time of deformation most of the total strain has to be assumed elastic. This assessment is supported by data published in [25]. For an LDPE similar to the sample presented in Figure 10, which was uniaxially stretched up to a total Hencky strain of 3 at an elongational rate of 1 s^−1^, a recoverable Hencky strain of 2.1 is reported. As a result of the distinctly shorter deformation times in case of the entrance flow in Figure 10, still larger recoverable portions for the uniaxial extension in the channel axis can be expected and, thus, the assumption of a mainly elastic extensional deformation in the entrance region of a die like that used for the measurements of Figure 10 is realistic.

#### 3.3.2. Extrudate Swell

**Figure 11:**
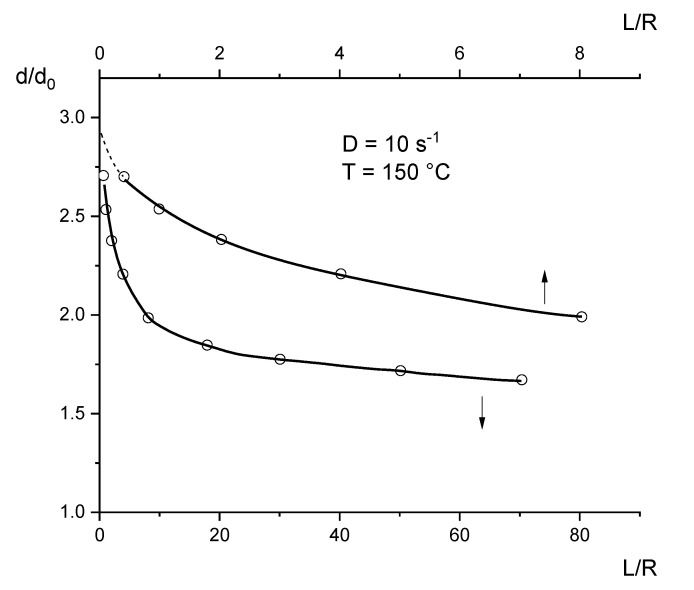
Extrudate swell $d/d_{0}$ as a function of the length to radius ratio L/R for an LDPE extruded at the apparent shear rate D=10 s^−1^ through a capillary with the radius R = 1.5 mm [25]. The upper curve and L/R-axis represent the extrudate swell for the smaller L/R at higher resolution.

The recoverable portion of the elongational flow in the entrance region of a die cannot directly be measured. However, it manifests itself in the extrudate swell attained after running through capillaries with small lengths minimizing the influence of shear and retardation processes within a die. Measurements of the extrudate swell from short circular capillaries with a diameter of 3 mm are reported for an LDPE in [25]. They were obtained after total recovery in a silicone oil bath and represent equilibrium values. Although the contraction ratios and the geometries of the dies used for the measurements of extrudate swell and extension in the entrance region are different, some qualitative conclusions from the extrudate swell presented in Figure 11 are of interest. For the commercial LDPE at an apparent shear rate of $D=10\;s^{-1},$ the extrudate swell $d/d_{0}$ decreases with the length to radius ratio L/R, as found for many polymers. The extrudate swell extrapolated to a capillary length of zero, which is related to the recoverable portion exerted by the entrance flow, attains a value of about 3. For a uniform uniaxial deformation of an incompressible fluid, this change in diameter would correspond to a recoverable Hencky strain of 2.2, at a first approximation. From this rough assessment, the assumption based on the short deformation times in the entrance region gets some support for the Hencky strain of ε = 2.5 in Figure 10, following on from the direct measurement of the velocity field in the entrance region of a slit capillary with LDV, is predominantly elastic. Thus, the extrudate swell occurring for short dies is strongly related to the recoverable elongation.

According to Figure 11, the extrudate swell decreases with increasing length of the capillary. As is well known, this effect is due to the time-dependent recovery of the elastic deformation of the melt previously exerted in the entrance region by passing the capillary. From the data in [25], it can be seen that the extrudate swell of the LDPE approaches a finite value at large L/R, which was extrapolated to lie at around 1.5. This feature of a constant extrudate swell for long dies has been reported in the literature for various polymer materials. This is due to normal stresses occurring during shear flow in a capillary, and relaxing when the melt is able to freely flow after exiting. Detailed information on extrudate swell and its dependence on experimental parameters and molecular structure of the polymer can be found in textbooks on rheology ([1,9], for example).

Direct measurements of elongational flow fields in the entrance region of a die and the analysis of their recoverable portions by discussing the extrudate swell obtained from orifices provide several insights. They throw a light on the vagueness of the fundamentals of common methods to determine elongational viscosities from the entrance flow to capillaries, which are in use for the modelling of processing operations with extensional components. Furthermore, recoverable elongations support a more thorough understanding of processing operations where the extrudate swell from short dies comes into play.

#### 3.3.3. Entrance Flow and Elongational Viscosity 

**Figure 12:**
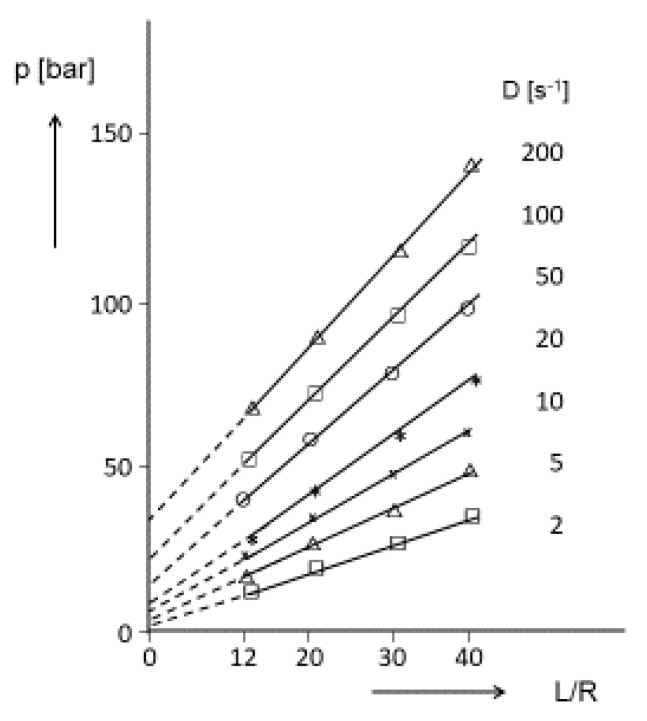
Extrusion pressure p as a function of the length to radius ratio L/R of a capillary for a commercial polystyrene at different apparent shear rates D.

The entrance flow is connected with some energy consumption, which results in a decrease of the amount of applied extrusion pressure available for the shear deformation of a melt within a capillary. This pressure loss can either be determined directly by monitoring the pressure drop of an orifice or by measuring the extrusion pressures at constant apparent shear rates as functions of L/R. An example of the last method for a commercial polystyrene is presented in Figure 12. This kind of plot was used by Bagley [26] many years ago, to determine the true pressure within a capillary for viscosity measurements. The pressure extrapolated to L/R = 0 is interpreted as the pressure loss, due to entrance effects. The obvious stretching flow in the entrance region of a die was the base for defining a tensile stress from the pressure drop. Using a simplified model of the entrance flow, Cogswell derived an expression for the elongational rate under the assumption of the velocity field for a power-law fluid. He arrived at an equation for the extensional viscosity as a function of pressure loss, apparent shear rate, shear viscosity, and power-law index [27]. Due to the lack of reliable physically based methods to determine elongational viscosities at rates relevant for processing, data calculated according to Cogswell´s model are frequently applied, and using the software of some commercial capillary rheometers, elongational viscosities as functions of elongational rate are provided routinely.

In addition to the inconsequence underlying the derivation of elongational rates from shear properties, some more doubts regarding the physical relevance of Cogswell´s equations come up from the direct measurement of a polyethylene melt in Figure 10 and the discussion of the extensional behavior in Section 3.3.1. From these considerations it is obvious that the deformation in the entrance region of a die at an apparent shear rate of 100 s^−1^ has to be assumed being predominantly elastic and, thus, the determination of elongational viscosities from entrance flow lacks a real physical base.

#### 3.3.4. Relevance of Extrudate Swell from Short Dies for Processing

A processing operation where short dies come directly into play is pelletizing. Pellets are essential for an effective feeding of the plasticizing units of nearly all kinds of processing equipment. Pelletizing is achieved by extruding the molten polymer through a punched disk and cutting the exiting strands with a rotating knife. This process should be as economical as possible and, thus, to minimize the extrusion energy, the thickness of the disk chosen is as small as possible, with respect to the mechanical stability. Therefore, the holes represent short dies and most of the energy consumption for flow is related to the entrance flow patterns. The entrance pressure losses exemplarily shown in Figure 12 reflect these features, and can attribute to assess the power necessary for the driving unit of the pelletizer. To obtain a maximum number of extruded strands, the distribution of holes on a disk should be chosen as dense as possible, but it has obviously to be avoided that the extrudates touch each other and glue together. As a result of the high outputs applied and the short processing times, the deformation at the entrance to the disk openings is mainly elastic and, thus, the extrudate swell is largely determined by the recoverable portion of the extensional flow. Therefore, the basic knowledge about the extensional behavior and its recoverable portion supports design and performance of the pelletizing process. Particularly, from the influence of molar mass distribution and long-chain branching on the recoverable elongation, conclusions can be drawn with respect to the extrudate swell of polymer melts from short dies.

Capillaries with small length to radius ratios are frequently used in fiber spinning. However, the role of extrudate swell is diminished by the stretching process often applied to the fibers when leaving the spinneret.

Nozzles with small L/R are found in extrusion units for fused deposition modelling (FDM), widely used in additive manufacturing. Here, the extrudate swell comes into play when the geometry of the layers has to be considered for the dimensional accuracy of the part generated [28]. In [28], only the extrudate swell according to shear is addressed, but, due to the nozzle geometries, an effect of the extensional flow in the entrance region and particularly of its recoverable portion should become significant. Due to the short distance between nozzle exit and substrate surface, in some cases the extrudate swell of the filament has not attained its equilibrium before fusion and solidification and, thus, some of the recoverable elongation is frozen, and gives rise to internal stresses of the manufactured item, which may influence mechanical properties and dimensional stability. Comprehensive investigations of these special features and numerical descriptions have not been available up to now, but they would be helpful for a more precise optimization of fused deposition modelling. The fundamental knowledge about recoverable extension could at least provide some hints to a better understanding of the processing performance.

#### 3.3.5. Extrudate Swell and Recoverable Elongation of Dispersed Polymer Melts

For many applications, polymers with fillers or polymer blends are used. Thus, it is of interest how extrudate swell and recoverable elongation of these materials may be related to each other. This topic is of particular interest for materials with dispersed phases of different deformability. Particles of minerals or carbon black, for example, are rigid under the conditions applied in polymer processing, but components like in polymer blends often exhibit viscoelastic properties, and can deform under external stresses. These different systems are discussed in the following section.

##### Polymers Filled with Rigid Particles

**Figure 13:**
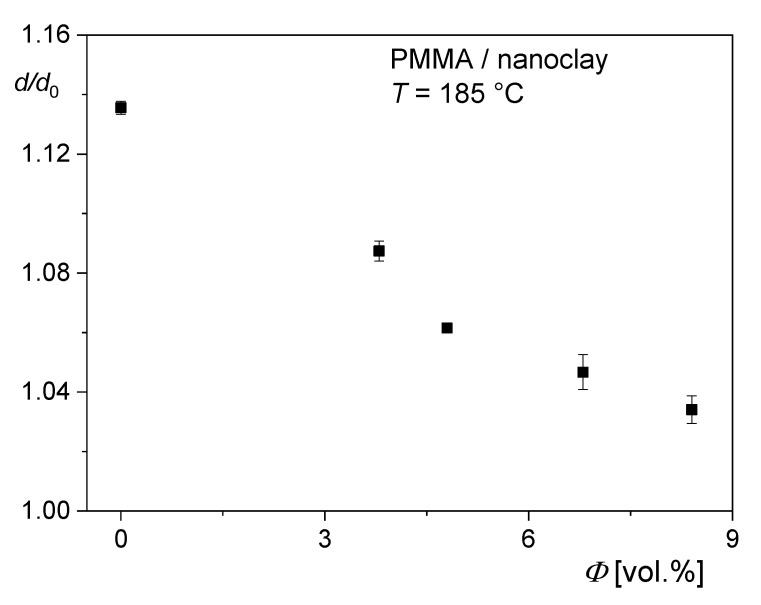
Extrudate swell $d/d_{0}$ of polymethylmethacrylate (PMMA) filled with nanoclay in dependence on the volume concentration *ϕ* at a wall shear stress of 90 kPa and T = 185 °C. The diameter of the die was $d_{0}$= 2.1 mm and its length L = 8 mm. d is the diameter of the strand completely annealed after extrusion [29].

Rheological investigations in shear on polymer melts filled with rigid particles are widely reported in the literature. Some of the results are compiled and discussed in [29]. For the extrudate swell, a decrease with increasing filler content is generally found. Fewer measurements exist on the influence of fillers on extensional properties and on the recoverable elongation, in particular. Very rare are investigations on recoverable elongation and extrudate swell of the same material. An example is presented for a polymethylmethacrylate (PMMA) filled with nanoclay of the montmorillonite type in [29]. The nanoclay used, the sample preparation, and the features of exfoliation and particle distribution are described in [30]. In Figure 13, the extrudate swell $d/d_{0}$ is presented for PMMA with various volume fractions of nanoclay. The length to radius ratio of the die was L/R = 7.6. Thus, it can be expected that the extrudate swell measured is representative for the recoverable portion of the elastic elongation exerted in the entrance region of the die, because the recovery during passing the short capillary can be assumed to be minute. The extrudate swell decreases with clay concentration supporting the picture of a simple replacement effect of the viscoelastic polymer by the rigid filler. The extrudate swell of the PMMA matrix is small in comparison to values known for other polymers (cf. the LDPE in Figure 11). This is due to the narrow molar mass distribution of the linear PMMA molecules. 

**Figure 14:**
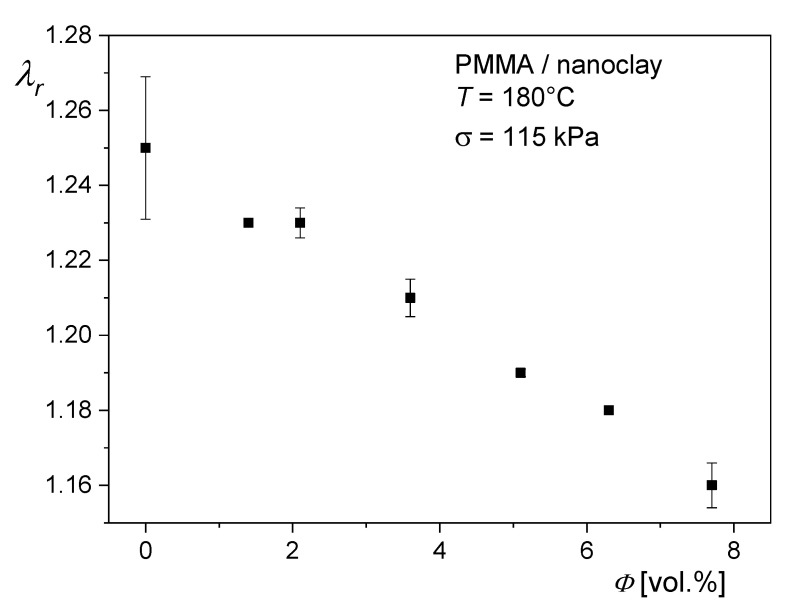
Recoverable stretching ratio $\lambda_{r}\;=\;l/l_{r}$ for the PMMA of Figure 13 as a function of the volume concentration *ϕ* of the nanoclay. The experiments were performed at a constant tensile stress of *σ* = 115 kPa and T = 180 °C. The stretching ratio *λ* = $l/l_{0}$ was 12, before recovery with the initial length of the sample $l_{0}$ = 25 mm [29].

The recoverable stretching ratio $\lambda_{r}$=$\;l/l_{r}$ of the PMMA filled with the same nanoclay is shown in Figure 14 as a function of the volume concentration *ϕ*. l is the length of the stretched sample before and $l_{r}$ the length after recovery. The samples were uniaxially elongated at the conditions given in the caption to Figure 14. The recoverable stretching ratio distinctly decreases with filler content, and supports the observations from unfilled polymers that the elastic components of entrance flow determine the extrudate swell from short dies. 

##### Immiscible Polymer Blends

**Figure 15:**
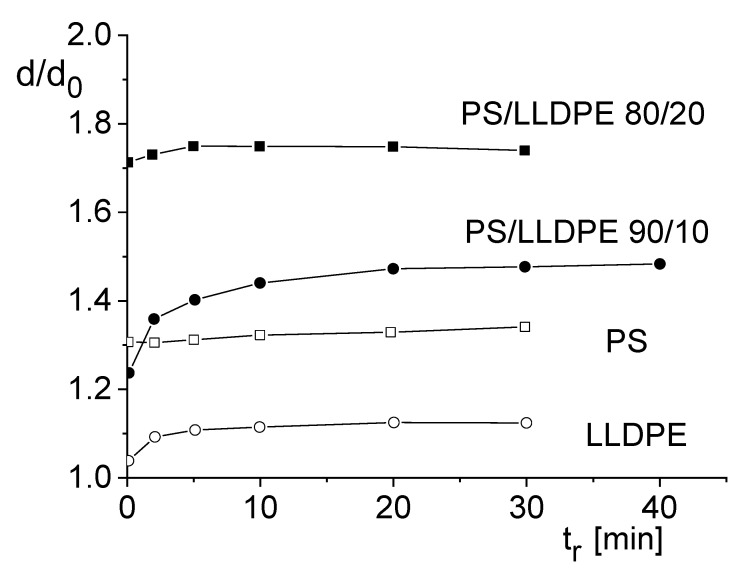
Extrudate swell $d/d_{0}$ as function of the annealing time $t_{r}$ in a silicone oil bath for two PS/LLDPE blends and their components. The diameter of the die was $d_{0}\;$= 2.1 mm and its length to radius ratio L/R= 7.6. The shear stress at the wall was $\sigma_{w}$= 40 kPa [29].

In polymer blends, the matrix and the dispersed phase also are viscoelastic materials. These properties result in a swell behavior completely different from that of polymer systems filled with rigid particles. In Figure 15 the extrudate swell for two blends of a commercial PS and LLDPE and that of the blend components immiscible with each other are presented. Equilibrium values were attained, as shown by the swell data as a function of the annealing time in a silicone oil bath. The length to radius ratio of the die was L/R = 7.6 and the shear stress at the wall $\sigma_{w}\;$= 40 kPa. Compared to the extrudate swell of PMMA filled with nanoclay in Figure 13, the most significant feature is that the extrudate swell of the PS/LLDPE blends lies above the swell of the PS matrix, although LLDPE exhibits a distinctly smaller swell than PS. The extrudate swell for PS/LLDPE 80/20 is higher than that of PS/LLDPE 80/10, indicating an effect related to the LLDPE phase.

**Figure 16:**
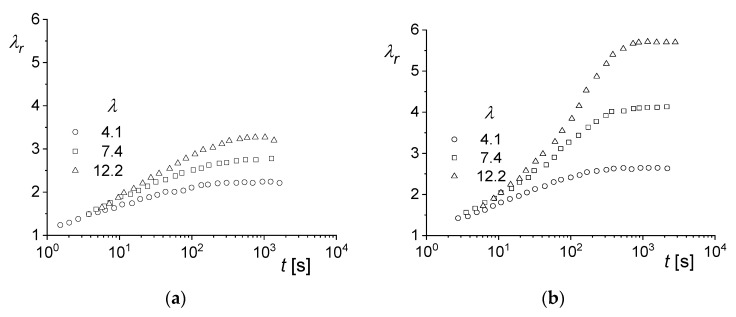
Recoverable stretching ratio $\lambda_{r}$ as a function of the recovery time t for (**a**) PS and (**b**) PS/LLDPE 85/15 after different stretching ratios λ. The samples were uniaxially elongated at $\dot{\varepsilon }$ = 0.1 s^−1^ and T= 170 °C [31].

As a result of the relatively short die, the extrudate swell is mainly determined by the stretching flow in the entrance region. Therefore, measurements of the recoverable elongations of the samples are of interest. In Figure 16, the recoverable elongations of the polystyrene matrix and the blend PS/LLDPE 85/15 after three different extensions are presented. As is well known and documented in Figure 2 for an LDPE, for example, the recoverable portion of the deformation increases with the previous total extension. Such a behavior is found for the PS matrix and the blend, but the increases for the blend are higher than for the matrix. Thus, the differences between matrix and blend are more pronounced at the larger stretching ratio of λ= 12.2 than at λ = 4.1. The increase of extrudate swell, going along with that of the recoverable elongation, is another indication of the decisive role of the elastic deformation in the entrance region for the extrudate swell from short dies.

**Figure 17:**
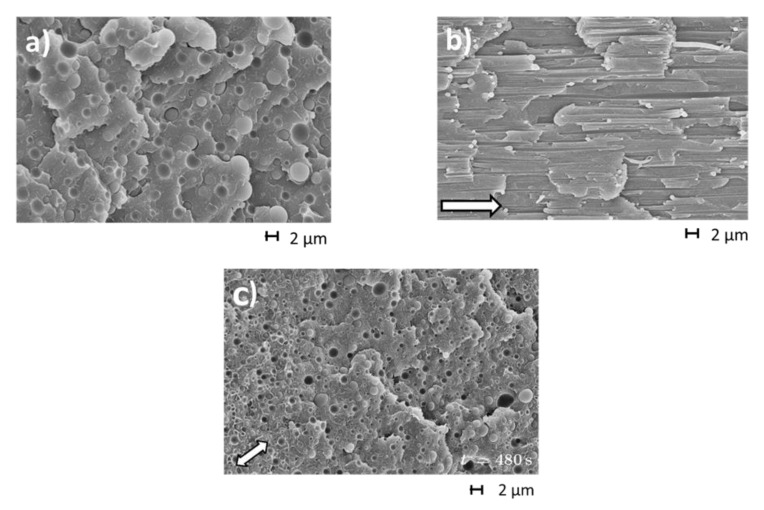
Scanning electron micrographs of the blend PS/LLDPE 85/15 (**a**) before stretching, (**b**) in the elongated state at λ=12 and (**c**) after recovery for 480 s at 170 °C. The sample was deformed at the extensional rate $\dot{\varepsilon \;}$= 0.1 s^−1^ and T = 170 °C. The arrows indicate the stretching direction [31].

The opposite influence of rigid fillers and viscoelastic blend components on elastic properties of the matrix has its origin in the deformability of the polymeric phase and its reversibility. Figure 17 shows electron micrographs of the PS/LLDPE 85/15 blend before elongation, in the state of deformation up to the stretching ratio of λ=12 and after total recovery. The minor phase LLDPE has a spherical shape in the initial state. At λ = 12, fibrils with a large aspect ratio are seen and after recovery droplets are found again. From these morphological investigations it is obvious that the stretching of the LLDPE phase and its recoil during recovery is the source of a recoverable elongation superimposed on the reversible deformation of the molecules of the PS matrix. This contribution becomes more pronounced the stronger the stretching of droplets into fibrils.

A comparison of the droplet sizes before deformation and after recovery reveals their reduction by the stretching process applied. This interesting feature indicating a decay of fibrils is beyond the scope of this paper, but it is comprehensively described in [32] and discussed in detail in [29]. 

## 4. Conclusions

The recoverable elongation, which can be measured in uniaxial elongation in dependence on a wide range of experimental parameters, is a rheological property not as frequently discussed as the elongational viscosity. Like other rheological quantities, the recoverable elongation is distinctly influenced by the molecular structure of polymers, and particularly by the molar mass distribution and long-chain branching, as documented in the literature. Some applications of polymeric materials are largely determined by their elastic behavior in extension, so it is worthwhile considering its role. Shrink films are one example. The recoverable portion of films attained during stretching in the molten state is frozen, and then released by heating above the melting or glass transition temperature in case of their application, as cover for various items. Fundamental knowledge about the dependence of the elastic portion of stretching on the molecular structure and external parameters can be used to support the development of foils for special packaging applications and of shrinkable tubes and sleeves used in demanding technical applications. 

The extrudate swell appearing in many processing operations based on extrusion has its origin in elastic deformations of polymer melts, due to shear and extension. Its elongational components come significantly into play for the swell from short dies of melts flowing through narrowing ducts. This was explicitly shown by an analysis of entrance flow of a commercial LDPE into a capillary. Using laser-Doppler velocimetry a broad distribution of elongational rates was found in the entrance region of a slit die at conditions typical of extrusion. A comparison with the results from well-defined extensional experiments at corresponding rates and elongations led to the conclusion of highly elastic extensions. This recoverable deformation is more pronouncedly reflected by the extrudate swell the shorter the time for recoil of the stretched molecules passing an attached capillary. Thus, the extrudate swell through an orifice is very closely related to the reversible portion of stretching flow in the entrance region.

These effects and insights play a role in processing operations where melts are extruded through short dies. An example is the pelletizing process widely used as the final manufacturing step for polymeric materials. For the optimized design of granulating plants, the knowledge of the energy loss due to entrance flow and of the extrudate swell occurring from the holes of a punched pelletizing plate can support the optimization of this process.

Another example is the extrusion step in fused deposition modelling (FDM). Due to dies with short lengths at the exit of the extrusion unit, the recoverable part of the extensional flow in the entrance region comes into play, and needs attention for comprehensive descriptions of this process in additive manufacturing, attaining more and more importance.

The dominating elastic portions of entrance flow throw a light on another feature of elongational rheology. The frequently applied method after Cogswell to calculate elongational viscosities in a range relevant to processing is based on pressure loss and viscous flow in the entrance region. Obviously, the method of deviating elongational viscosity from entrance flow patterns being mainly elastic lacks any realistic base, however. 

The correlation between the prominent elastic deformation in the entrance flow of narrowing ducts manifested by the extrudate swell from short capillaries and the directly measured recoverable elongation in uniaxial extension is supported by investigations on a particle filled polymer and a polymer blend. The extrudate swell of a polymer melt filled with nanoclay decreased as a function of concentration in parallel to the recoverable elongation. For PS/LLDPE blends, the extrudate swell and the recoverable elongation as well were found to be larger than for the components. Morphological investigations of the blend samples in different states of stretching showed an extension of the dispersed LLDPE drops to fibrils and their regression to spherical shapes after stress release. This effect superposes the recoil of the stretched matrix molecules, and contributes essentially to the recoverable deformation of the sample.
